# Internal Maxillary Artery Pseudoaneurysm: A Near Fatal Complication of Seemingly Innocuous Pharyngeal Trauma

**DOI:** 10.1155/2011/241375

**Published:** 2011-09-22

**Authors:** M. P. Hennus, L. Speleman

**Affiliations:** ^1^Department of Pediatric Intensive Care, Wilhelmina Children's Hospital, University Medical Center Utrecht, P.O. Box 85090, 3508 AB Utrecht, The Netherlands; ^2^Department of Pediatric Otorhinolaryngology, Wilhelmina Children's Hospital, University Medical Center Utrecht, P.O. Box 85090, 3508 AB Utrecht, The Netherlands

## Abstract

A 2-year-old boy presented with persistent pain and oral blood loss after falling with a toothbrush in his mouth. Initial routine inspection of the oropharynx showed no abnormalities. Recurrent blood loss instigated a reinspection under general anesthesia revealing the head of the toothbrush embedded in the nasopharynx. The toothbrush was removed without problems but several hours later a near fatal rebleeding occurred, requiring aggressive fluid resuscitation. Subsequently, the patient was transferred to our pediatric intensive care unit for further evaluation and treatment. CT angiography (CTA) showed a pseudoaneurysm of the internal maxillary artery which was successfully coiled, and further recovery was uneventful. Pediatric pharyngeal trauma is a common entity with rare, but potentially life-threatening, complications. In case of pharynx lesions, bleeding, and persistent pain, flexible endoscopy by an otolaryngologist is mandatory. In case of persistent bleeding vascular imaging is essential with CTA being a reliable alternative for the more invasive angiography.

## 1. Introduction

Pediatric pharyngeal trauma is relatively common in young children and usually caused by stick-like objects. Fortunately, most injuries are confined to mucosal and/or dermal surfaces and recovery is often uneventful without need for interventions. However rare, potentially devastating complications do occur, even in seemingly innocuous injuries. The first and most common complication is development of free air in the neck (subcutaneous emphysema) and/or chest (pneumomediastinum), which can lead to acute airway obstruction [1–4]. Furthermore, infections like retropharyngeal abscesses or mediastinitis may occur [1–4]. Vascular damage however, poses the greatest risk and is potentially life threatening [3, 5–9]. This paper not only illustrates how a common and seemingly innocuous injury can prove to be nearly fatal, it also emphasizes the need for vascular imaging in case of persistent blood loss after pharyngeal trauma.

## 2. Case Presentation

**Figure 1:**
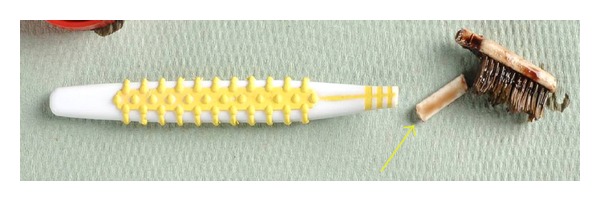
On the right, pieces of the head of the toothbrush (yellow arrow) immediately after removal from the patients oro-nasopharyngeal junction. On the left, the base of the toothbrush which the patient was found holding after his fall.

A previously healthy 2-year-old boy presented at the emergency department of a local hospital with persistent pain and oral blood loss after an unwitnessed fall. Immediately afterwards, he was found crying with only the base of a toothbrush in his hand. The head of the previously intact toothbrush was missing. During initial inspection of the oral cavity, the bleeding had stopped and no abnormalities were seen by the otolaryngologist. Although the parents repeatedly expressed their concern about their son's persisting abnormal behavior and the still missing head of the toothbrush, the patient was discharged with instructions to return to the hospital in case of a fever and/or recurrent blood loss. That same night, the boy started vomiting fresh blood and was taken to the hospital by ambulance. Again the bleeding had stopped spontaneously but the persistent character of the blood loss initiated a reinspection of the oronasopharynx under general anesthesia by an otolaryngologist. This revealed the missing head of the toothbrush (Figure 1) lodged horizontally in the lateral walls of the nasopharynx with its sharp end embedded in the left lateral pharyngeal wall. It was removed without problems, making a small lesion visible in the left pharyngeal wall. The patient was successfully extubated and transferred to the pediatric ward.

Several hours afterwards however, he started vomiting large amounts of blood again, rapidly leading to progressive lethargy, tachycardia, and severe hypotension. Because of life-threatening hypovolemic shock with a hemoglobin level of 2.9 mmol/L, large amounts of intravenous saline and an unmatched blood transfusion were given. Crash reintubation followed, and during a second reinspection of the oro- and nasopharynx the bleeding again had stopped spontaneously. The site of the previous blood loss could not be identified. A Bellocq tampon was left in place and given the hemodynamic instability, and the still unknown origin of the massive bleeding the patient was transferred to our pediatric intensive care unit. 

**Figure 2:**
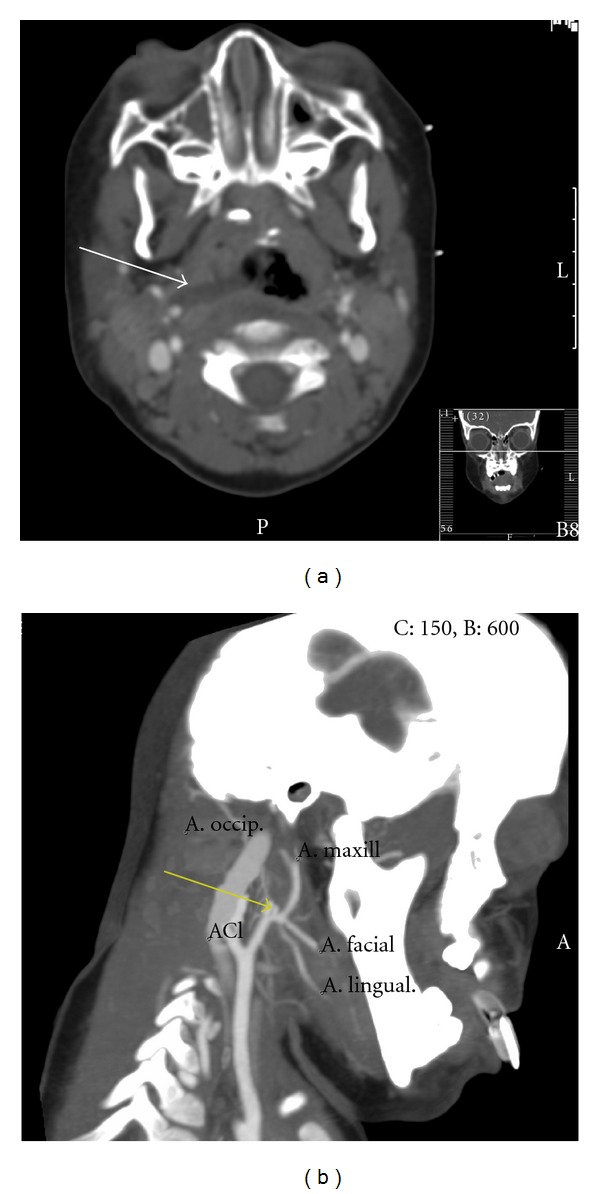
CT angiography of the head and neck showing a hematoma shaped like the head of the toothbrush in the right nasopharynx (white arrow) and the aneurysmatic dilatation of the origin of the internal maxillary artery (yellow arrow).

**Figure 3:**
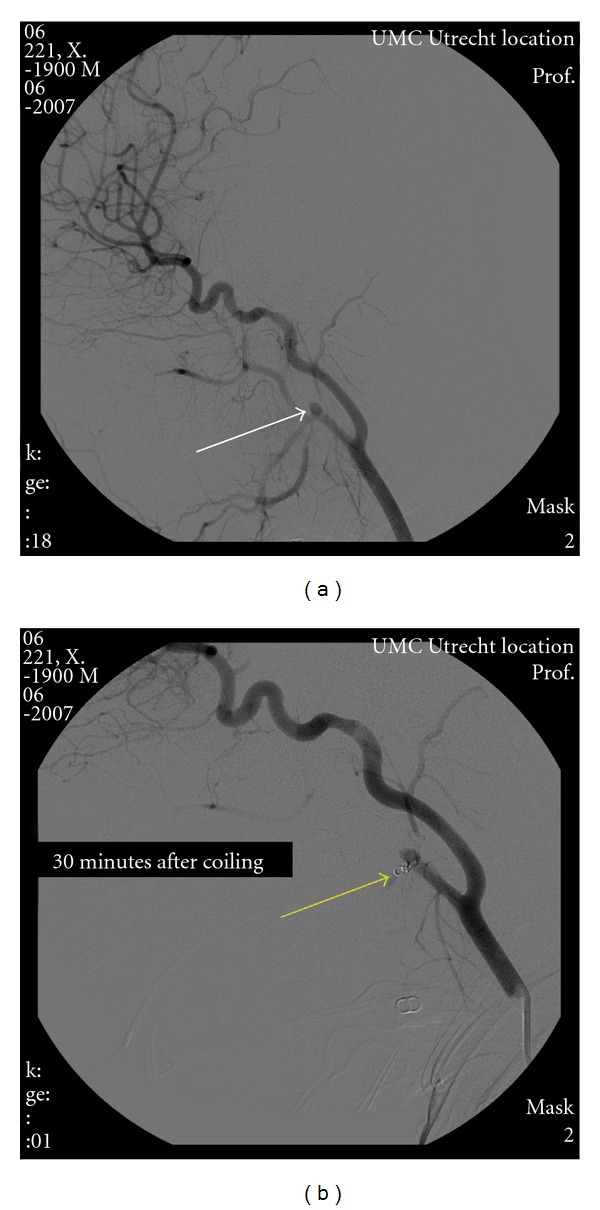
Conventional angiography of the head and neck showing the pseudoaneurysm at the base of the internal maxillary artery (white arrow) and the coil (yellow arrow) closing the pseudoaneurysm successfully.

On admission, the patient was hemodynamically stable without signs of persisting blood loss, and invasive interventions were withheld. Mechanical ventilation was continued, antimicrobial therapy was started, and the Bellocq tampon was to be removed 48 hours later. That night however, during routine endotracheal suctioning, the patient started to cough inducing pulsatile oral blood loss suspect for an arterial vascular injury. The bleeding was successfully controlled, and subsequently a CT angiography (CTA) of the head and neck was performed. This showed a long, hypodense region in the right nasopharynx suspect for a hematoma as well as an aneurysmatic dilatation of the origin of the right internal maxillary artery (Figure 2). The subsequently performed conventional angiography confirmed the presence of an internal maxillary artery pseudoaneurysm, approximately 2 cm after the bifurcation of the arteria carotis communis which was successfully coiled (Figure 3). Two days later the Bellocq tampon was removed without problems, and a week after the initial fall, the patient could be extubated. He was discharged just 4 days after extubation. Clinical followup shortly after discharge and 1 year after the incident revealed no sequelae. 

## 3. Discussion

Our patient survived a near fatal bleeding from an internal maxillary artery pseudoaneurysm resulting from a seemingly innocuous injury after a fall with a toothbrush in his mouth. The incidence of pharyngeal trauma in young children is probably underreported. The initial trauma is usually unwitnessed and patients only present in case of persistent pain, dysphagia, or blood loss. Although the injuries are mostly confined to mucosal and/or dermal surfaces, seemingly innocuous injuries can result in severe blood loss and/or neurological deficits.

A careful medical history, including the time and cause of the incident as well as the initial and presenting complaints, is essential. Furthermore, persisting parental concern, especially when based on persisting complaints or abnormal behavior of their child as in this case, should always alert the physician. Routine inspection of the oral cavity and pharynx, even when meticulously performed, is not sufficient in pharyngeal trauma with persistent complaints. Here endoscopic inspection of the whole oro-, naso-, and hypopharynx is warranted. Unfortunately, vascular lesions can develop, even in the absence of a visible pharyngeal lesion: our patients' pseudoaneurysm was located on the right side of the pharynx and not on the left where the sharp, broken end of the toothbrush had left a small mucosal lesion. Compression of the carotid artery between the foreign body and the transverse process of a cervical vertebrae, namely, can result in an intimal tear in the vessel. This so-called “shearing effect” can lead to a pseudoaneurysm, dissection, and/or thrombosis of the vessel itself with the subsequent risk of migration of (a part of) the thrombus to the cerebral vessels [5]. 

Neurological complications develop in the first 3 to 60 hours after the initial trauma. This lucid period is believed to correlate with the time needed for formation and propagation of the thrombus [1]. Anticoagulants are recommended in the presence of a pseudoaneurysm and/or thrombus but only when the risk of an ischemic infarct outweighs the risk of a hemorrhagic infarct [1, 3, 4, 6]. Blood loss due to dissection of a vessel or pseudoaneurysm, as seen in our patient, also occurs within these first 60 hours. When refraining from admitting a patient, it is mandatory to provide parents or caregivers with necessary information concerning this lucid period, possible complications, and instructions how to act when problems do occur.

Although the incidence of vascular injuries following pharyngeal trauma is less than 1% [2, 3], the sequelae can be disastrous [7–9]. Unfortunately, prognostic (risk) factors enabling early detection of vascular injuries before the onset of neurological signs and symptoms are still lacking [2]. Imaging studies are warranted in recurrent or persistent blood loss. The “gold standard” in diagnosing internal carotid artery damage is angiography, an invasive and rather time-consuming procedure with a small, but not neglectable, risk of neurological complications. A CTA offers a quick and effective alternative, which can be followed by an (intervention) angiography when a vascular injury is suspected [2, 10].

In conclusion, pediatric pharyngeal trauma is a common entity with rare, but potentially severe, complications. Vascular injuries can develop even in the absence of visible pharyngeal lesions. A thorough history and physical examination including inspection of the oro-naso-hypopharynx are warranted. Flexible endoscopy by an otolaryngologist is mandatory in case of pharynx lesions, bleeding, and persistent pain. Vascular imaging is essential in persistent blood loss, with CTA offering a reliable alternative to the more invasive angiography. Given the possible lucid period in a vascular injury between the initial trauma and the development of sequelae, it is essential, when refraining from hospital admission, to provide parents or caregivers with the necessary information concerning these complications and with instructions how to act when problems do occur.
